# Integrin α6 promotes esophageal cancer metastasis and is targeted by miR-92b

**DOI:** 10.18632/oncotarget.14259

**Published:** 2016-12-27

**Authors:** Gang Ma, Chao Jing, Furong Huang, Xukun Li, Xiufeng Cao, Zhihua Liu

**Affiliations:** ^1^ The State Key Laboratory of Molecular Oncology, National Cancer Center/Cancer Hospital, Chinese Academy of Medical Sciences and Peking Union Medical College, Collaborative Innovation Center for Cancer Medicine, Beijing, China; ^2^ Department of Oncological Surgery, The Affiliated Nanjing 1st Hospital, Nanjing Medical University, Nanjing, China

**Keywords:** integrin α6, esophageal cancer, metastasis, microRNA-92b

## Abstract

Tumor invasion and metastasis is responsible for the poor prognosis of esophageal squamous cell carcinoma (ESCC); therefore, exploring the mechanisms by which malignant cells disseminate, spread and flourish in secondary sites, as well as translating the bench results to clinical practice are in urgent need. Previous reports showed that integrin α6 increases in ESCC specimens and its dysregulated spatial localization correlates positively with the unfavorable outcome of ESCC patients. Here, we clarify that integrin α6 promotes invasion and metastasis of ESCC cells *In vitro* and *in vivo*. Mechanistically, decreased integrin α6 attenuates motility of malignant cells partially through deactivating Akt pathway, which is essential for ESCC cells motility. Moreover, integrin α6 serves as a genuine target of miR-92b in suppressing ESCC motility. Our results for the first time describe that miR-92b/integrin α6/Akt axis controls the motility of ESCC, thereby providing a promising diagnosis or therapeutic option.

## INTRODUCTION

Esophageal squamous cell carcinoma (ESCC) is a heavy health burden; in 2015, 375,000 new deaths were in Chinese ESCC patients [1]. The poor outcome of ESCC patients is primarily attributed to metastasis [2]. Extensive studies have revealed dozens of proteins and non-coding RNAs implicating in the invasion and metastasis of ESCC cells. However, our understanding of the invasion-metastasis cascade of ESCC is far from thorough because this is a complicated process.

Integrins are a group of heterodimers consisting of one α subunit and one β subunit. To date, 18 α subunits and 8 β subunits have been identified in mammalians and they form 24 heterodimers [3]. Among these heterodimers, integrin α6 binds to integrin β1 or β4 respectively, and plays critical roles under physiological and pathological conditions [4]. Specifically, expression of integrin α6 elevates across a wide range of cancers. In ESCC, for example, expression of this α subunit is dramatically higher in malignant specimens than that of adjacent normal tissues [5]. One recent study described that aberrant spatial distribution of integrin α6 correlates with the poor outcome of ESCC patients [6]. However, mechanisms of integrin α6 in ESCC progression remain obscure.

MicroRNAs are a group of non-coding RNAs regulating gene expression at the post-transcriptional level [7]. In distinct types of cancer, microRNAs serve as pro- or anti-metastasis regulators [7]. Several microRNAs have been found to modulate the metastasis of ESCC [8–10] and the inhibitory role of miR-92b, for example, in invasion-metastasis cascade of ESCC was described previously in our lab [11]. However, the functions of miR-92b are not fully understood.

In this study, we demonstrate that integrin α6 promotes invasion and metastasis of ESCC cells at least partly dependent on the activated Akt pathway. Moreover, integrin α6 acts as the direct target of miR-92b, the microRNA which dramatically suppresses the motility of ESCC cells. The newly found miR-92b/integrin α6/Akt axis is a critical regulator of ESCC metastasis.

## RESULTS

### Integrin α6 positively correlates with the lymph node metastasis of ESCC

**Figure 1:**
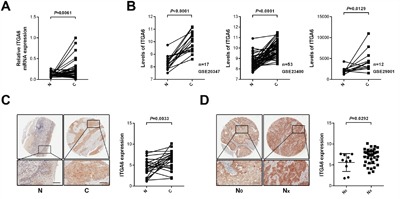
Expression of integrin α6 in ESCC specimens **A**. The mRNA levels of integrin α6 in ESCC tissues and paired normal tissues were detected using qPCR, showing that integrin α6 in malignant esophageal tissues was significantly higher than that of normal ones (n = 28). GAPDH was used as the internal control. Paired *Student's t*-test was used to analyze data. **B**. Analysis of three expression datasets from GEO demonstrated that integrin α6 increased in ESCC specimens relative to that of adjacent normal counterparts. **C**. Immunohistochemistry assay showed that integrin α6 elevated in cancerous esophageal tissues. Representative IHC image is shown. **D**. ESCC cells with the increased integrin α6 expression were prone to invade lymph nodes. N_0_ (n = 10) means primary ESCC lesions without lymph node metastasis and N_X_ suggests all primary ESCC specimens with lymph node metastasis (N_1_-N_3_, n = 39). Scale bars in C and D represent 100 μm.

In order to reveal the clinical relevance of integrin α6 in ESCC, we first assessed the expression of integrin α6 in ESCC specimens using qPCR. Results showed that the expression of integrin α6 significantly increased in the malignant tissues relative to the paired adjacent normal tissues (n = 28, Figure 1A), which was in line with three reported results deposited in Gene Expression Omnibus (GSE20347, GSE23400 and GSE29001) (Figure 1B). Integrin α6 showed higher expression in the cancerous tissues compared with that of the adjacent normal tissues (Figure 1C). More importantly, analysis of IHC results revealed that integrin α6 positively associated with the lymph node metastasis other than gender, age, tumor size and TNM classification of ESCC (Figure 1D and Table 1). These results together demonstrate that expression of integrin α6 is increased in ESCC tissues, and this overexpression probably promotes the metastasis of ESCC cells.

**Table 1:** Association between clinicopathologic characteristics and ITGA6 expression

Total	*n*	Low expression	High expression	*P*-value*
	48	23	25	
**Age (Years)**				
Mean	62 (range:44-80)			
≤60	20	9 (39%)	11 (44%)	
>60	28	14 (61%)	14 (56%)	0.732
**Sex**				
Male	40	19 (83%)	21 (84%)	
Female	8	4 (17%)	4 (16%)	0.897
**Tumor Size (cm^3^)**				
≤24	25	12 (52%)	13 (52%)	
>24	23	11 (48%)	12 (48%)	0.990
**TNM classification**				
I/II(I-II included)	41	21 (91%)	20 (80%)	
III(II-III included)	7	2 (9%)	5 (20%)	0.268

* *P*-values are from χ^2^ test and P<0.05 was considered statistically significant.

### Integrin α6 promotes motility and metastasis of ESCC cells *in vitro* and *in vivo*

**Figure 2:**
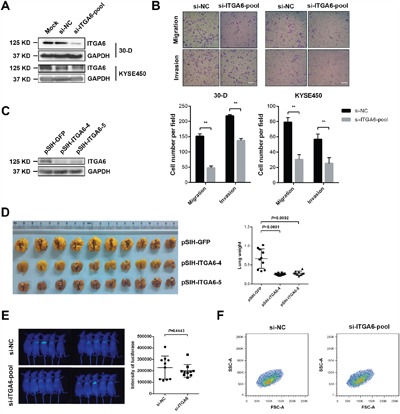
Decreased level of integrin α6 inhibits invasion and metastasis of ESCC cells **A**. Representative result of integrin α6 after being knocked down using a pool of three siRNAs (100 nM in total) against this integrin in 30-D or KYSE-450 cells respectively. This assay was repeated twice. **B**. Reduced integrin α6 impaired the migration and invasion of both 30-D and KYSE-450 cells *in vitro*. At least three fields were selected randomly to count the penetrated cells. Scale bars in the images represent 400 μm. This assay was repeated three times independently. **C**. ESCC 30-D cells with stably decreased integrin α6 were constructed and the expression was detected to evaluate the efficiency of these two shRNAs. ShRNA against GFP was engineered and used as the negative control. **D**. Experimental metastasis assay showed that inhibition of integrin α6 dramatically impeded the pulmonary metastasis of ESCC cells. 5×10^5^ of cells with decreased integrin α6 and the control cells were injected into immunocompromised mice via tail veins respectively. Lungs were harvested, weighed and compared between the control and the treated groups (n = 10 in each group). **E**. Inhibition of integrin α6 did not affect the pulmonary arrest of ESCC cells *in vivo*. The injected cells were pre-labelled with luciferase and the luciferase activities were detected within 24 hr after the injection (n = 10 in each group). **F**. Loss of integrin α6 did not influence the cellular size relative to these control cells. Specific siRNAs against integrin α6 and the scramble oligos were delivered into 30-D cells respectively and the cell size from the two groups were compared 48 hr after the transfection. This assay was repeated three times independently.

We next examined the effect of integrin α6 on the motility and metastasis of ESCC cells. We introduced a pool of siRNAs against integrin α6 into 30-D and KYSE-450 cells respectively and found that reduced integrin α6 significantly impaired the migration and invasion of these treated cells compared with that of control cells (Figure 2A and 2B). Integrin α6 was reduced in 30-D cells using two shRNAs, the expression of which was detected using immunoblots (Figure 2C).

Then, *in vivo* experiment showed that 30-D cells with stably reduced integrin α6 form far fewer overt metastases relative to the control cells (Figure 2D and Supplementary Figure 1). However, decreased integrin α6 did not alter cell size or hamper pulmonary arrest of 30-D cells. There was no significant difference in luciferase intensity between the control and the knockdown group (Supplementary Figure 2). These results indicated that this integrin probably influenced the other steps of invasion-metastasis cascade (Figure 2E and 2F). These data demonstrate that integrin α6 promotes metastasis of ESCC cells.

### Integrin α6-Akt pathway stimulates motility of ESCC cells

**Figure 3:**
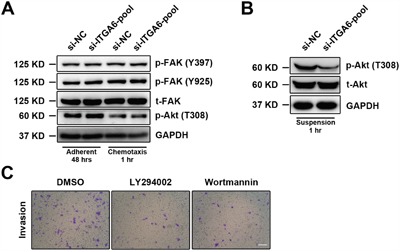
Integrin α6 regulates cellular motility of ESCC through Akt pathway **A**. Under the either adherent or chemotaxis condition integrin α6 did not affect FAK or Akt phosphorylation at the indicated time points. The representative results of three independent assays are shown. **B**. Loss of integrin α6 significantly decreased the level of phosphorylated Akt (T308) when cells were suspended in the 6-well dishes pre-treated with poly-HEMA for 1 hr. This assay was repeated three times independently. **C**. Two inhibitors of Akt activation impaired invasion of the treated 30-D cells relative to the control counterparts. The scale bar in the image represents 400 μm. This assay was repeated three times independently.

Akt pathway is aberrantly activated in esophageal cancer and FAK pathway is involved in motility of ESCC. In order to study how integrin α6 promoted metastasis of ESCC cells, we then assessed the expression of p-Akt (T308) and p-FAK (Y397 and Y925) in 30-D cells transfected with siRNAs against integrin α6 under adherent condition (48 hr) or chemotaxis condition (1 hr); however, no detectable changes were observed in these three phosphorylated proteins (Figure 3A). We then coated the 6-well culture dishes with poly-HEMA and incubated 30-D cells in suspension for 1 hr. Interestingly, less integrin α6 reduced p-Akt (T308) in 30-D cells compared with those control cells (Figure 3B). We finally examined the invasiveness of 30-D cells pre-treated with LY294002 or wortmannin (4 hr), and the penetrated cells were fewer than the control cells (Figure 3C). Collectively, these data indicate that integrin α6-Akt pathway is essential for the invasion of ESCC cells.

### Integrin α6 is targeted by miR-92b in ESCC cells

**Figure 4:**
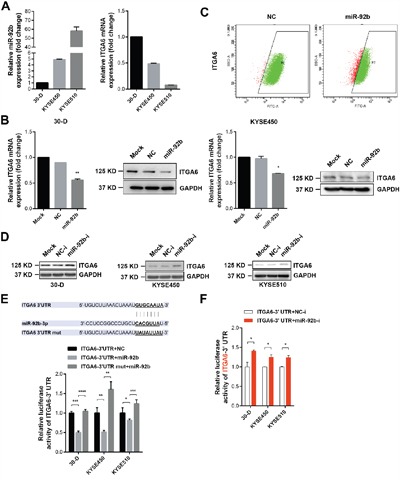
Integrin α6 is a genuine target of miR-92b in ESCC cells **A**. The expression of integrin α6 correlated reversely with miR-92b level in ESCC cell lines. GAPDH acted as the internal control of the qPCR assays. This assay was repeated twice. **B**. Transient transfection of miR-92b and the scramble oligo respectively into 30-D or KYSE-450 cells decreased the expression of integrin α6 at both mRNA and protein level. This assay was repeated twice. **C**. Flow cytometry assay demonstrated that increased level of miR-92b also reduced the membrane expression of integrin α6 in 30-D cells. This assay was repeated three times independently. **D**. Deletion of miR-92b in ESCC cells dramatically increased the expression of integrin α6. This assay was repeated twice. **E**. The predicted binding site of miR-92b in the 3’UTR of integrin α6 was cloned into the downstream of luciferase and the mutation was constructed as well. Dual luciferase reporter assay showed that miR-92b bound directly to the 3’UTR of integrin α6. This assay was repeated three times independently. **F**. Inhibition of endogenous miR-92b enhanced the luciferase activity of plasmids containing wild type binding site of miR-92b. This assay was repeated three times independently.

We previously established one pair of cell subpopulations (30-U/D) with distinct motility capacity from KYSE-30 cells and found that miR-92b dramatically suppressed the metastasis of 30-D cells (GSE67510) [11]. We first examined the expression of integrin α6 in HET-1A, 30-D, KYSE-450 and KYSE-510 cells and showed higher ITGA6 expression in ESCC cell lines. Moreover, expression of integrin α6 was relatively higher in 30-D cells (stronger motility capacity) than that in KYSE-450 and KYSE-510 cells (weaker motility capacity) (Supplementary Figure 3). More importantly, miR-92b in 30-D, KYSE-450 and KYSE-510 cells correlated inversely with integrin α6 (Figure 4A). We transfected 30-D and KYSE-450 cells with miR-92b mimic and scramble oligo respectively, and then detected the mRNA and protein level of integrin α6. As a result, integrin α6 decreased at both mRNA and protein level in response to miR-92b mimic transfection (Figure 4B). Of note, miR-92b diminished the membrane expression of integrin α6 in 30-D cells (Figure 4C). Specific inhibitor against miR-92b (miR-92b-i) and the control oligo (NC-i) were also introduced into 30-D, KYSE-450 and KYSE-510 cells respectively, and immunoblots showed that integrin α6 increased considerably (Figure 4D).

Moreover, we cloned the presumed binding site of miR-92b from 3’-UTR of integrin α6 into the downstream of luciferase in pISO plasmid (ITGA6-3’UTR), and then mutated the binding site (ITGA6-3’UTR-mut) (Figure 4E). We transfected ITGA6-3’UTR or ITGA6-3’UTR-mut with miR-92b mimic and scramble oligo (NC) respectively, showing that luciferase activity in the cells transfected with ITGA6-3’UTR was reduced significantly by miR-92b compared with the control cells whereas luciferase activity in the cells transfected with ITGA6-3’UTR-mut was restored relative to the cells transfected with ITGA6-3’UTR (Figure 4E). Additionally, when we co-transfected ITGA6-3’UTR with miR-92b-i or NC-i respectively, luciferase activity increased as the result of inhibition in endogenous miR-92b (Figure 4F). These data together demonstrate that ITGA6 expression is regulated by miR-92b at the posttranscriptional level.

### MiR-92b inhibits motility of ESCC cells partially through integrin α6

**Figure 5:**
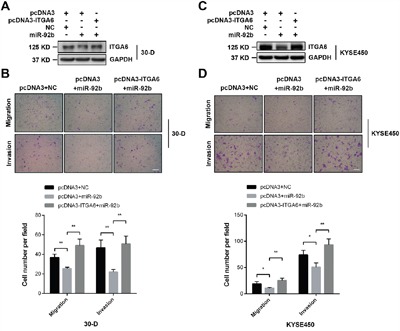
Integrin α6 is a functional target of miR-92b in ESCC cells **A** and **B**. Restored expression of integrin α6 in 30-D cells pre-treated with miR-92b promoted migration and invasion of these cells *in vitro*. **C** and **D**. Re-expression of integrin α6 could rescue the motility of 30-D cells *in vitro*, which was impeded by miR-92b. The scale bars in the images represent 400 μm. The statistical results were from at least three randomly selected fields. All these results were from three independent assays.

As we have described that miR-92b could dramatically suppress invasion-metastasis cascade of ESCC [11], we then examined whether integrin α6 was implicate in this regulation. We restored integrin α6 expression in 30-D cells transfected with miR-92b mimic (Figure 5A), and observed that increased level of integrin α6 could cushion the inhibitory effect of miR-92b on migration and invasion (Figure 5B). Likewise, restored integrin α6 expression in KYSE-450 cells transfected with miR-92b mimic also enhanced motility of these transfected cells *in vitro* (Figure 5C and 5D). Therefore, integrin α6 is a functional target of miR-92b in suppressing motility of ESCC cells.

## DISCUSSION

Integrin α6 stimulates metastasis of breast cancer, colorectal cancer, prostate cancer, and non-small lung adenocarcinoma [12–15]. Data presented here demonstrate a strong association between the increased expression of integrin α6 and the lymph node metastasis of ESCC. We also provide *in vitro* and *in vivo* evidence showing that reduced integrin α6 strikingly undermines the motility and pulmonary metastasis of ESCC cells. Mechanistically, loss of integrin α6 reduces the activated Akt (T308) under the suspended condition, and suppression of Akt activation using LY294002 and Wortmannin hampers the motility capacity of ESCC cells. Moreover, we verify that integrin α6 is a functional target of miR-92b, this microRNA which has been described to inhibit the invasion-metastasis cascade of ESCC cells. Accordingly, miR-92b/integrin α6/Akt axis is crucial in motility and metastasis of ESCC cells.

Our findings support the other studies about the roles of integrin α6 in ESCC [5, 16]. Y. Tanaka and colleagues found that integrin α6 increases in malignant esophageal tissues and IHC analysis suggested that this integrin correlates with the lymph node metastasis [5]. J. Kwon and colleagues reported that integrin α6 sustains the proliferation and motility of ESCC cells *in vitro*, and they further used antibody against integrin α6 (GoH3) to significantly inhibit the tumor growth of ESCC cells in mice [16]. Likewise, our results confirm the pro-motility ability of integrin α6 in ESCC cells and, more importantly, showing that integrin α6 is essential for establishing overt lung metastases without influencing pulmonary arrest of the cancerous cells in the compromised mice. However, the exact function of integrin α6 has not been fully clarified in this study; thereby, it is not clear that absence of integrin α6 inhibits formation of overt metastases through either disabling the initiation of proliferation or hampering the rapid growth of colonized ESCC cells. Considering that integrin α6β1 and α6β4 can also be secreted in exosomes by tumor cells to build niche in favor of lung-specific metastases [17], it is critical to our future work in delineating the mechanistic details of integrin α6 in ESCC metastasis.

Another limitation of this study is that we did not elucidate how integrin α6 regulates activation of Akt. This is partly due to the strong adhesion ability of 30-D cells, which leads to cell attachment even in the culture plates pre-treated with polyHEMA and subsequent Akt activation within a relatively short time (Data not shown). Therefore, we can only detect the reduced p-Akt (T308) within 1 hr in the integrin α6-silenced ESCC cells without adhesion-elicited disturbance. However, one study on breast cancer demonstrates that integrin α6β4 relays invasion signals through recruiting Shc to further stimulate Akt activation [18]. Thus, we speculate that integrin α6 probably activates Akt in the same manner because integrin α6β4, instead of integrin α6β1, is found in ESCC tissues [16].

In this study, we also prove that integrin α6 is an additional genuine target of miR-92b, which undoubtedly deepens our understanding of how miR-92b functions in each step of the invasion-metastasis cascade of ESCC cells and provides an opportunity of applying miR-92b in diagnosis or treatment of ESCC invasion and metastasis. Notably, the function of integrin α6 is different from that of integrin αV. For example, miR-92b overexpression or integrin αV deletion hampered the pulmonary arrest of ESCC cells [11], whereas knockdown of integrin α6 did not affect this process (Figure 2E). Mechanistically, integrin αV or α6 promoted motility of ESCC cells through different signaling pathways: the former affected FAK-Rac1 signaling pathway [11] while the latter probably deactivated Akt pathway without involving into FAK phosphorylation (Figure 3A). These preliminary results suggest the distinct roles of integrin α6 and αV in the metastatic cascade of ESCC cells. Moreover, we did not observe the reduced Akt activation in the suspended 30-D cells with overexpressed miR-92b (Data not shown), which is probably because miR-92b-mediated loss of integrin α6 is not enough to change Akt pathway. Collectively, future study on how integrin α6 assists proliferation of the settled ESCC cells in lungs is required.

In conclusion, we corroborate that integrin α6 could promote metastasis of ESCC cells for the first time. We also find that reduced integrin α6 inhibits the formation of overt metastases in lungs instead of suppressing pulmonary arrest. Akt pathway is crucial for viability, proliferation and motility of ESCC cells; loss of integrin α6 deactivates Akt of the malignant esophageal cells in suspension. However, the roles of activated Akt in the proliferation of the ESCC cells *in vivo* is still not clear. More importantly, miR-92b directly targets integrin α6, which increases the possibility of miR-92b as a promising treatment option. Collectively, miR-92b/integrin α6/Akt axis is a critical point for the diagnosis and treatment of ESCC metastasis.

## MATERIALS AND METHODS

### Cell culture and reagents

ESCC cell lines (KYSE30, KYSE450 and KYSE510) were provided by Dr. Y. Shimada (Kyoto University, Kyoto, Japan) [19]. These cells and two ESCC sublines (30-U/D) derived from KYSE-30 cells [11] were cultured in RPMI1640 with 10% FBS, streptomycin (100 mg/ml) and penicillin (100 U/ml). HEK293T cells were obtained from ATCC (Manassas, VA, USA) and cultured in DMEM with 10% FBS, streptomycin (100 mg/ml), and penicillin (100 U/ml). All cells were cultured under humanized condition (37°C, 5% CO_2_). STR analysis was carried out to authorize ESCC cells.

Akt inhibitors, LY294002 and Wortmannin, were purchased from Cell Signaling Technology (Danvers, MA, USA) and the cells were pre-treated for 4 hr before the transwell assay. PolyHEMA was obtained from Sigma-Aldrich (Shanghai, China) and coat of 6-well dishes with 2 ml polyHEMA (10 mg/ml in ethanol) was repeated twice and these treated dishes were air-dried in the sterilized safety cabinet before addition of cells.

### Immunohistochemistry

ESCC tissue microarray (HEso-Squ127lym-01) was purchased from Shanghai Outdo Biotech Co., Itd (Shanghai, China). Fresh ESCC tissue specimens and matched normal adjacent tissues were collected from patients undergoing surgery in Chinese Academy of Medical Sciences Cancer Hospital. All experiments performed on tissue samples were approved by the ethical committee of the Chinese Academy of Medical Sciences Cancer Hospital.

The paraffin-embedded ESCC tissue array (HEso-Squ127lym-01) was stained with primary antibody against ITGA6 (1:50) (Supplementary Table 1) and visualization was used Vectastain ABC kit and DAB chromagen from Vector Laboratories (Burlingame, CA, USA). The mounted specimens were then analyzed using ImageScope system (GE, USA).

### RNA, clones and quantitative PCR

RNA from both cultured cells and tissue samples was extracted using Trizol (Invitrogen). RNA was reversely transcribed to cDNA according to the instruction of Quantscript RT kit (Tiangen, Beijing, China). The predicted binding site of miR-92b from integrin α6 3’-UTR was cloned into pISO, which was a generous gift from Dr. D. P. Bartel of Massachusetts Institute of Technology (Cambridge, MA, USA). Mutation of this binding site was constructed using KOD-Plus-Mutagenesis Kit (TOYOBO, Japan). All constructs were verified by sequencing (SinoGenoMax, Beijing, China).

Quantitative PCR was performed using SYBR Premix Ex Taq™ II (TaKaRa, Japan) on Step-one plus real-time PCR system (Applied Biosystems, CA, USA). GAPDH was used as the internal control for mRNAs and U6 for miR-92b, respectively. Quantitative PCR results were analyzed using 2^-ΔΔCt^ method. Primers are listed in Supplementary Table 2.

### Immunoblots

Cells were harvested under adherent, chemotaxis or suspension condition at the indicated time points using lysis buffer (10 mM Tris-HCl, 150 mM NaCl, 5 mM EDTA, 1% TritonX-100, and 0.25% sodium deoxycholate, pH=7.4) containing phosphatase and protease inhibitors (Roche) and stored in -80°C refrigerator. The following SDS-PAGE and blots were performed according to the standard procedure. The primary antibodies used in immunoblots are listed in Supplementary Table 1.

### Transwell assay

*In vitro* migration and invasion assays were performed as previously described [11]. During the assay, no LY294002 or Wortmannin was added into the medium. At least three fields were randomly acquired by Nikon Biophot (Japan) equipped with camera and software LAS v4.6. The number of the penetrated cells was represented as mean ±SD.

### Luciferase reporter assay

Luciferase activity was detected using Dual-Luciferase Reporter Assay (Promega, WI, USA) 24 hr after the pISO plasmids containing wild type or mutated binding site of miR-92b were transfected with miR-92b mimic, inhibitor or scramble oligos respectively into cells. Data were represented as mean ±SD from four parallel wells.

### Transfection and transduction

In order to knockdown the expression of integrin α6, three siRNAs against this integrin and scramble oligo were ordered from Integrated DNA Technologies (IA, USA). We used HiPerFect (Qiagen) to deliver a pool of the three siRNAs and scramble oligo into cells, respectively (100 nM).

We designed shRNA oligos (Supplementary Table 2) against integrin α6 and cloned them into pSIH-puro. Next, we used Lipofectamine 2000 (Invitrogen) to transiently transfect HEK293T cells with pSIH-ITGA6-4/5 with the package vectors, and harvested mature lentivirus as described previously [11]. Thawed lentivirus was added into 30-D cells with polybrene (6 μg/ml), and puromycin (1 μg/ml) was used to get stable clones.

### Flow cytometry

After being transfected with siRNAs against integrin α6 and scramble oligos respectively, 30-D cells were harvested and sent to analysis using flow cytometry. Forward scatter (FSC) was used to compare the cell volume of the treated 30-D cells with that of the control counterparts.

### Animal experiments

The equal amount of the treated 30-D cells with silenced integrin α6 expression and the control cells (5×10^5^ cells in 100 μl PBS) were injected into tail veins of the immunocompromised mice, respectively. Lungs of these recipient mice were obtained 12 weeks after injection and immediately weighed. The protocol was approved by the ethical committee of the Chinese Academy of Medical Sciences Cancer Hospital.

### Statistical analysis

All assays except animal and IHC experiments were performed at least twice independently. Exclusion criteria was established before analyzing IHC results. Paired or two-tailed *Student's t*-test was used in analyzing data, which were presented as mean ± SD. In the graphs, *, **, and *** demonstrate *P*< 0.05, 0.01 and 0.001, respectively.

## SUPPLEMENTARY MATERIALS FIGURES AND TABLES


