# Supplementary material for: Integrin α6 promotes esophageal cancer metastasis and is targeted by miR-92b
Source: Oncotarget. 2016 Dec 27;8(4):6681–90. doi: 10.18632/oncotarget.14259 (PMC5351662; doi:10.18632/oncotarget.14259)
Supplement: Supplementary file 1 [file oncotarget-08-6681-s001.pdf]

Integrin α6 promotes esophageal cancer metastasis and is targeted by miR-92b

SUPPLEMENTARY FIGURES AND TABLES

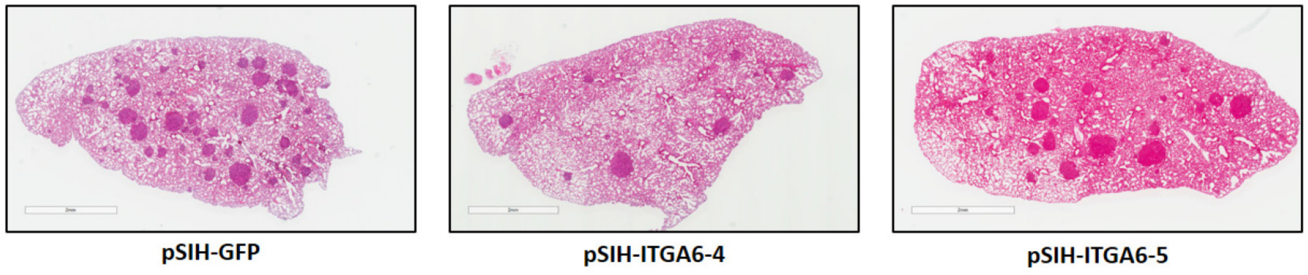

Supplementary Figure 1: H&E stain of the lungs from the control and the integrin α6 knockdown group. Fewer metastatic nodules could be observed in the lungs of the integrin α6 silenced mice than these counterparts from the control mice. The bars in the images indicate 2 mm.

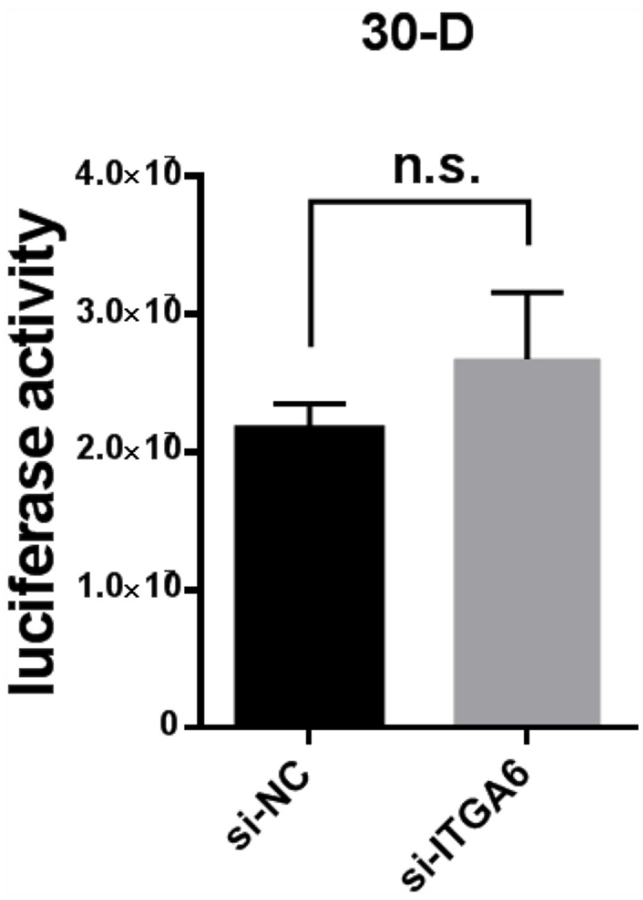

Supplementary Figure 2: Reduced integrin α6 does not affect the luciferase activity. The specific siRNAs (100 nM in total) against integrin α6 and the scramble oligos were delivered into 30-D cells, respectively. Around 20 hr later, the luciferase activity were detected using Luciferase Assay Kit (Promega, WI, USA).

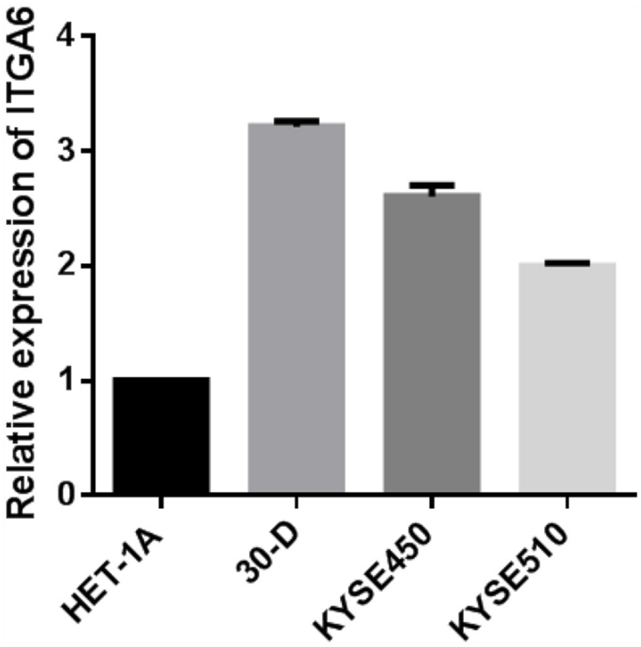

Supplementary Figure 3: Integrin α6 expression is higher in ESCC cell lines than that in normal esophageal cell line. The mRNA level of integrin α6 was detected by RT-qPCR. The data was analyzed using $2^{-\Delta\Delta Ct}$ method.

Supplementary Table 1: Primary antibodies used in this investigation

Primary antibody	catalog	Vendor	Application
ITGA6	3883-1	Epitomics	WB
ITGA6	NBP1-85747	NOVUS	IHC
ITGA6	561893	BD	FC
GAPDH	G8795	Sigma	WB
FAK Antibody Sample Kit	9330	Cell Signaling	WB
Phospho-Akt (Thr308)	2965	Cell Signaling	WB
Akt (pan)	4685	Cell Signaling	WB

Supplementary Table 2: List of primers and oligos used in this investigation

Application	Sequence
**Clone**	
ITGA6 3'UTR	F: TTGAGCTCAGAGTGACTACACACAGTACG
	R: TTGCTAGCTTATTAAAACAACTCTCTCTGGTAC
**microRNA reverse transcription**	
miR-92b	CTCAACTGGTGTCGTGGAGTCGGCAATTCAGTTGAGGGAGGCCG
**qPCR detection**	
miR-92b	F: ACACTCCAGCTGGGTATTGCACTCGTCCCG
	R: TGGTGTCGTGGAGTCG
ITGA6	F: GCTGGTTATAATCCTTCAATATCAATTGT
	R: TTGGGCTCAGAACCTTGGTTT
GAPDH	F: TGCACCACCAACTGCTTAGC
	R: GGCATGGACTGTGGTCATGAG
U6	F: CTCGCTTCGGCAGCACA
	R: AACGCTTCACGAATTTGCGT
**Mutation**	
ITGA6 UTR mut	F: AATCACGTTATGAAGGTGATGTTGCCATCCTAC
	R: TAGTTTAAGACAGCACTTTCCGTTGAAACACAACT
**shRNA oligos**	
ITGA6 sh-4 target	GTTTCTGGATTTCATACTGTAA
ITGA6 sh-5 target	ATCCCGGCCTGTGATTAATATT
GFP shRNA target	CGAGAAGCGCGATCACATG
